# Quantitative and Microscopic Assessment of Compatible and Incompatible Interactions between Chickpea Cultivars and *Fusarium oxysporum* f. sp. *ciceris* Races

**DOI:** 10.1371/journal.pone.0061360

**Published:** 2013-04-16

**Authors:** Daniel Jiménez-Fernández, Blanca B. Landa, Seogchan Kang, Rafael M. Jiménez-Díaz, Juan A. Navas-Cortés

**Affiliations:** 1 College of Agriculture and Forestry, University of Córdoba, Campus de Excelencia Internacional Agroalimentario ceiA3, Córdoba, Spain; 2 Institute for Sustainable Agriculture (IAS), Spanish National Research Council (CSIC), Córdoba, Spain; 3 Department of Plant Pathology and Environmental Microbiology, The Pennsylvania State University, University Park, Pennsylvania, United States of America; Freie Universität Berlin, Germany

## Abstract

**Background:**

Fusarium wilt caused by *Fusarium oxysporum* f. sp. *ciceris*, a main threat to global chickpea production, is managed mainly by resistant cultivars whose efficiency is curtailed by *Fusarium oxysporum* f. sp. *ciceris* races.

**Methodology:**

We characterized compatible and incompatible interactions by assessing the spatial-temporal pattern of infection and colonization of chickpea cvs. P-2245, JG-62 and WR-315 by *Fusarium oxysporum* f. sp. *ciceris* races 0 and 5 labeled with ZsGreen fluorescent protein using confocal laser scanning microscopy.

**Findings:**

The two races colonized the host root surface in both interactions with preferential colonization of the root apex and subapical root zone. In compatible interactions, the pathogen grew intercellularly in the root cortex, reached the xylem, and progressed upwards in the stem xylem, being the rate and intensity of stem colonization directly related with the degree of compatibility among *Fusarium oxysporum* f. sp. *ciceris* races and chickpea cultivars. In incompatible interactions, race 0 invaded and colonized ‘JG-62’ xylem vessels of root and stem but in ‘WR-315’, it remained in the intercellular spaces of the root cortex failing to reach the xylem, whereas race 5 progressed up to the hypocotyl. However, all incompatible interactions were asymptomatic.

**Conclusions:**

The differential patterns of colonization of chickpea cultivars by *Fusarium oxysporum* f. sp. *ciceris* races may be related to the operation of multiple resistance mechanisms.

## Introduction

Chickpea (*Cicer arietinum* L.) is a major source of human food and animal feed, and one of the world’s most important pulse crops after dry beans (*Phaseolus vulgaris* L.) and dry peas (*Pisum sativum* L.) [1]. Chickpeas are grown throughout tropical, subtropical and temperate regions of the world [1]. Fusarium wilt, caused by the soilborne fungus *Fusarium oxysporum* Schlechtend.: Fr. f. sp. *ciceris* (Padwick) Matuo & K. Sato, is one of the most important diseases limiting chickpea production worldwide. Annual yield losses from this disease have been estimated to range from 10 to 15%. However, locally Fusarium wilt epidemics can cause 100% loss under disease-favorable conditions [2], [3], [4].

Fusarium wilt in chickpea is best managed using resistant cultivars [5]. However, the effectiveness of this management strategy can be curtailed by the high pathogenic variability in *F. oxysporum* f. sp. *ciceris* populations [6]. Two pathotypes (yellowing and wilting) and eight pathogenic races (races 0, 1A, 1B/C, 2, 3, 4, 5, and 6) have been described to date [7], [8], [9]. The yellowing pathotype induces progressive foliar yellowing with vascular discoloration and late plant death, while the wilting pathotype causes fast and severe chlorosis, flaccidity, vascular discoloration, and early plant death. For both syndromes, the subterranean tissues of affected plants show no external symptoms.

Pathogenic races of *F. oxysporum* f. sp. *ciceris* differ in geographic distribution. Races 0, 1B/C, 5, and 6 are found in the Mediterranean Basin and California (USA) [3], [6], [9], [10]. Race 1A has been reported in the Indian subcontinent [2], California, and the Mediterranean Basin [6], [9], [10], whereas races 2 and 3 have been reported in Ethiopia, India, and Turkey [2], [11], [12] and race 4 has only been reported in Ethiopia and India [2], [12]. Races 0 and 1B/C induce the yellowing syndrome, whereas races 1A through 6 cause the wilting syndrome [6], [9]. Knowledge of the geographical distribution of *F. oxysporum* f. sp. *ciceris*-races is critical to disease management, because individual races vary in their interaction with differential chickpea lines as well as in the amount of inoculum needed to induce a given amount of disease in susceptible chickpeas [13], [14].

Our understanding of the cellular basis of host-pathogen interactions underlying vascular wilt diseases has been much improved due to the use of confocal laser scanning microscopy (CLSM) and fluorescent protein-tagged pathogen isolates. This approach has allowed ‘*in situ*’ time-lapse imaging of plant-pathogen interactions, thus avoiding concerns associated with fixing plant tissues for light and electron microscopy observations [15], [16]. Thus, use of CLSM has provided better understanding of the infection process of *F. oxysporum* in multiple hosts: i) the ability of *F. oxysporum* f. sp. *cubense* race 4 to directly penetrate epidermal cells of banana roots, and the role of the epidermal cells of root caps and elongation zone as potential sites of ingress [17]; ii) the root meristem of emerging lateral roots and primary root tips of *Arabidopsis thaliana* are infection courts for a wilting *F. oxysporum* isolate, regardless the level of resistance of the host ecotypes [18]; iii) the pattern of *Medicago trunculata* colonization by *F. oxysporum* f. sp. *medicaginis* is similar between susceptible and tolerant lines [19]; iv) differential virulence of *F. oxysporum* f. sp. *phaseoli* isolates on bean is directly correlated with the speed of xylem vessels colonization [20]; vi) resistant and susceptible melon lines are differentially colonized by *F. oxysporum* f. sp. *melonis* race 1.2 [21]; or vii) the infection of tomato plants by *F. oxysporum* f. sp. *lycopersici* is initiated by development of a hyphal network on the upper part of taproot, followed by the growth of hyphae towards the elongation zone, lateral roots and root apices [22].

Pathogenic races of *F. oxysporum* f. sp. *ciceris* differ significantly in the amount of inoculum needed to induce a given amount of disease in susceptible chickpeas [13], [14]. For example, the inoculum density of the least virulent race 0 needs to be ∼100 times greater than that of the highly virulent race 5 in order to cause the same amount of disease [13], [14]. Characterization of the underlying cause of differential virulence by different races will help understand how different chickpea genotypes interact with pathogenic races of *F. oxysporum* f. sp. *ciceris*. However, a few studies that have characterized the Fusarium wilt/chickpea pathosystem focused mainly on compatible interactions [23], [24], [25]. Therefore, additional studies are needed for better understanding the mechanisms underlying incompatible interactions.

In this present research, we hypothesized that the nature of *F. oxysporum* f. sp. *ciceris* races influences the processes of infection and colonization of resistant chickpeas by the pathogen, and addressed this hypothesis by determining the preferential infection court and quantitatively assessing the spatial-temporal patterns of colonization in both compatible and incompatible interactions with host cultivars differing in the level of resistance to *F. oxysporum* f. sp, *ciceris* races. To support this objective, isolates of two races (0 and 5) were first transformed with a ZsGreen fluorescent protein [18]. Then, one transformant of each race was selected based on growth characteristics, pathogenicity, and virulence phenotypes in comparison with the corresponding wild parent isolates. Finally, three cultivars that differentially respond to these races were inoculated to describe and quantify the pattern and dynamics of colonization.

## Materials and Methods

### Growth, Storage, and Genetic Transformation of *F. oxysporum* f. sp. *ciceris* Isolates

Two *F. oxysporum* f. sp. *ciceris* isolates (Foc-7802, race 0 and yellowing pathotype and Foc-W6-1, race 5 and wilting pathotype) were transformed with a construct encoding the ZsGreen fluorescent protein using the procedures described by Khang et al. [26]. Coding ZsGreen protein fragment was cloned in *Xba*I-*Hin*dIII sites of pBHt2 [27] to produce the binary vector SK2241. *Agrobacterium tumefaciens* strain EHA105 [28] was used to transform the isolates. Six and 11 transformants were obtained from Foc-7802 and Foc-W6-1, respectively. All transformants were grown on *Fusarium* minimal medium [29] amended with hygromycin B (50 µg ml^−1^). To check for the successful expression of ZsGreen, single-spored cultures of transformants were examined with a confocal laser microscope (Nikon TE 2000-S, Melville, NY, USA). Four transformants of each race were then chosen for subsequent phenotypic characterization.

The wild-type strains and their transformants were deposited in the culture collection of the Department of Crop Protection, Institute for Sustainable Agriculture (IAS-CSIC), Córdoba, Spain. All cultures were single-spored before long-term storage in sterile soil tubes at 4°C and in 35% sterile glycerol in water at −80°C. Active cultures were started by placing small aliquots of soil cultures onto a plate containing fresh potato dextrose agar (PDA; Difco Laboratories, Detroit, MI) and incubating for 4 days at 25°C and a 12-h photoperiod of a mixture of fluorescent white and near-UV light at 36 µE m^–2^ s^–1^.

### Phenotypic Characterization of Transformants

Colony morphology, mycelial growth rate, pathogenicity, and race phenotype of the selected transformants were compared with those of the corresponding wild-type strains. To compare for colony morphology and mycelial growth rate, a PDA plug of actively growing culture was placed onto PDA plates and incubated at 25±1°C and a 12-h photoperiod (fluorescent white and near-UV light at 36 µE m^–2^ s^–1^). Three replicates (plates) for each of the transformants and wild-type strains were arranged in a completely randomized design. The radial mycelial growth (RMG) was determined daily by measuring the length of four radii. The radial growth rate (RGR) was calculated by the slope of the linear regression of the mean colony radius over time. Mycelial pigmentation, the shape of colony margin, and any feature that looked different from wild-type strains were recorded.

Pathogenicity and race phenotypes of the transformants were assessed by inoculating chickpea cultivars that differ in their reaction to races 0 and 5, including cvs. P-2245 (susceptible to both races), PV-1 (susceptible to race 0 and resistant to race 5), JG-62 (resistant to race 0 and susceptible to race 5), and WR-315 (resistant to both races). Inocula were prepared in Erlenmeyer flasks containing 400 g of a cornmeal-sand mixture (CMS) infested by the fungus, and mixed with a sterile potting mix as described before [14]. Plants were grown and infected at 10^5^ cfu g soil mix^−1^ in 15-cm diameter clay pots (four plants per pot) in a walk-in growth chamber adjusted to 25±2°C and a 14-h photoperiod (fluorescent white light at 360 µE m^–2^ s^–1^) for 42 days. There were four replicated pots per isolate arranged in a completely randomized design. Incidence (*I,* 0 to 1 scale), and severity of foliar symptoms [*S*, rated on a 0 to 4 scale according to the percentage of affected foliage (0 = 0%, 1 = 1−33%, 2 = 34−66%, 3 = 67−100%, 4 = dead plant)] were assessed on individual plants at 2- to 3-day intervals. Disease intensity index (DII) was obtained using the following: DII = (*I* × *S*)/4 [14]. Disease progress curves were obtained from the accumulated DII over time from the date of inoculation.

### Cytological Characterization of Compatible and Incompatible Interactions via Confocal Laser Microscopy

Two ZsGreen-tagged transformants of races 0 and 5, named as F11 and F93, respectively, were used to infect chickpea cvs. P-2245, JG-62, and WR-315, which differ in disease reaction to races 0 and 5. Inocula were mostly microconidia obtained from cultures grown in Erlenmeyer flasks containing 100 ml of potato dextrose broth (PDB) (250 g of potato, 20 g of D-glucose, 1,000 ml of deionized water) as described before [30]. The liquid cultures were filtered through eight layers of sterile cheesecloth, and the conidia suspensions were adjusted with sterile deionized water to a concentration of 10^5^ conidia ml^−1^. Chickpea seeds were germinated at 25±1°C in sterile sand for 24 h and seedlings with about 1-cm-long radicle were transferred into sterile plastic pots (6-cm diameter, 100-ml volume) containing 80 ml of the conidial suspension. Seedlings serving as control were transferred to sterile distilled water in the same pots. There were two seedlings per pot for each of transformant/chickpea cultivar/sampling time combinations (see below). Pots with plants were placed in an orbital shaker adjusted at 120 rpm located in a walk-in growth chamber adjusted to same conditions described above for 18 days. The hydroponic cultures were supplemented daily with a complete Hoagland nutrient solution [31] as required to maintain nutrient availability and compensate for water loss. Disease reactions were assessed daily for the severity of foliar symptoms (*S*) as described above. The experiment was arranged in a completely randomized design, and it was repeated twice.

Inoculated and control seedlings were sampled daily from day 1 to 4 after inoculation and at a 2-day interval thereafter, up to 18 days after inoculation. At each sampling date, four plants (two pots, two plants each) were collected from each treatment. The entire surface of the tap and lateral roots of each plant was observed under epifluorescence light and confocal laser microscopy. Images were acquired by excitation with 488 nm argon laser and using a ZsGreen specific filter (515–530 nm) for detection of fluorescence emitted by the pathogen and plant autofluorescence at wavelength of 550–590 nm. Transverse sections from each of the sampled seedlings, *ca*. 400 µm thick, were used for analyses of seedling colonization. Sections were made using a hand microtome from fragments of each of the following tissues: tap root zones (apex, intermediate, upper), hypocotyl (zone immediately before the insertion of cotyledons), epicotyl (zone just above insertion of cotyledons) and one to fifth stem internodes. Sections were observed at 200x (Plan Fluor ELWD 20x objective), 400x (Plan Fluor ELWD 40x objective), and 600x (Plan Ado VC 60x objective) (Nikon Inc.). The extent of xylem vessel colonization was assessed quantitatively using images obtained at 200x. Colonization was estimated by the incidence of vascular colonization (IcVC) estimated as the proportion of xylem cells with fungal structures, and intensity of vascular colonization (ItVC) estimated as the percentage of the xylem vessels lumina filled with fungal structures. For each seedling and sampled area combination, the vascular cylinder was divided into four identical portions (blocks) and fungal colonization incidence and intensity were assessed in 20 vessels per block covering the full surface of the vascular cylinder.

### Data Analyses

The final radius of the fungal colony after 9 days of incubation (RMG_final_), and the radial growth rate (RGR) estimated by the slope of the regression line of the radial growth over time, were determined for each of the transformants and wild type strains. Data were subjected to standard analysis of variance using the GLM procedure of SAS 9.2 (SAS Institute Inc., Cary, NC, USA). The estimated values of both parameters for each of the transformants were compared with those of the wild-type strains using the Dunnett’s contrast at *P*<0.05.

Disease reaction of chickpea plants were characterized by three variables associated with the disease progress curve: i) Incubation period (IP), established as the time in days to display initial symptoms; ii) Disease intensity index (DII_final_) = disease severity observed at the final date of disease assessment; and iii) SAUDIIPC = standardized area under the DII progress curve calculated by trapezoidal integration standardized for the duration of disease development in days [32]. The estimated values of these parameters were statistically compared as described above. The effects of experimental treatments on the IcVC and ItVC were analysed by standard analysis of variance with the GLM procedure of SAS. In addition, the effect of different combinations of selected experimental treatments was assessed by single degree of freedom contrasts at *P*<0.05.

## Results

### Generation and Characterization of Transformants of Two *Fusarium oxysporum* f. sp. *ciceris* Races

Two *F. oxysporum* f. sp. *ciceris* strains (Foc-7802, race 0 and yellowing pathotype and Foc-W6-1, race 5 and wilting pathotype) were transformed with a gene encoding the ZsGreen fluorescent protein. Mycelial growth rates of the wild-type strains and selected transformants, and the disease reactions in chickpea cvs. JG-62, P-2245, PV-13, and WR-315 are shown in Table 1. The four transformants of *F. oxysporum* f. sp. *ciceris* race 0 and the wild-type strain showed similar (*P*≥0.05) mean final radial mycelial growth (RMG_final_) and RGR values, and virulence as determined by the IP, DII_final_ and SAUDIIPC, except for the followings: IP of disease reactions induced by isolates F3 and F8 in cvs. P-2245 and PV-1, and isolate F12 in cv. PV-1, were significantly lower (*P*<0.05) than those induced by the wild type strains (Table 1). Moreover, of the four transformants of race 5, F73 and F94 showed mean values of RMG_final_ and RGR significantly lower (*P*<0.05) than those of the wild-type strain. Also, the IP of disease reaction induced by transformants F83, F93, and F94 in cv. JG-62 was significantly (*P*<0.05) lower compared with that induced by the wild-type strain (Table 1). Based on the results described above, transformants F11 of *F. oxysporum* f. sp. *ciceris* race 0 and F93 of *F. oxysporum* f. sp. *ciceris* race 5 were selected for further experiments in this study as the transformed isolates showing overall pathogenic and morphological characteristics closest to those of the wild type strains.

**Table 1:** Characterization of two isolates of *Fusarium oxysporum* f. sp. *ciceris* (races 0 and 5) transformed with the ZsGreen fluorescent protein.

Isolates^a^	Mycelial growth^b^		Elements of the disease progress curve^c^											
			DII_final_				SAUDIIPC				IP			
	RMG_Final_ (mm)	RGR (mm/h)	P-2245	JG-62	PV-1	WR-315	P-2245	JG-62	PV-1	WR-315	P-2245	JG-62	PV-1	WR-315
***F. oxysporum*** ** f. sp. ** ***ciceris*** ** race 0**														
F3	36.3	0.18	1	0.00	1	0.00	0.76	0.00	0.81	0.00	16.37*	–d	16.50*	–
F8	36.3	0.18	1	0.00	1	0.00	0.75	0.00	0.84	0.00	16.87*	–	16.50*	–
F11	35.8	0.18	1	0.00	1	0.00	0.71	0.00	0.81	0.00	20.12	–	18.87	–
F12	35.9	0.18	1	0.00	1	0.00	0.73	0.00	0.83	0.00	19.40	–	17.43*	–
Foc-7802(wt)	36.2	0.18	1	0.00	1	0.00	0.71	0.00	0.84	0.00	20.00	–	20.30	–
***F. oxysporum*** ** f. sp. ** ***ciceris*** ** race 5**														
F73	29.3*	0.14*	1	1	0.00	0.00	0.87	0.90	0.00	0.00	13.75	15.62	–	–
F83	37.3	0.17	1	1	0.00	0.00	0.89	0.88	0.00	0.00	12.53	12.60*	–	–
F93	36.6	0.17	1	1	0.00	0.00	0.90	0.90	0.00	0.00	13.00	12.68*	–	–
F94	31.5*	0.15*	1	1	0.00	0.00	0.88	0.87	0.00	0.00	13.07	14.28*	–	–
Foc-W6-1(wt)	40.2	0.19	1	1	0.00	0.00	0.86	0.90	0.00	0.00	12.93	15.56	–	–

a These strains were genetically transformed with the ZsGreen fluorescent protein according to Khang et al. (2006); (wt) indicates wild-type strains.

b RMG_F_ = Radial Mycelial Growth Final, assessed by the average value of the fungal colony radius reached after 9 days growing at 25±1°C and a photoperiod of 12-h of a mixture of fluorescent white and near-UV light at 36 µE m^–2^ s^–1^. RGR = Radial Growth Rate: slope of linear regression model that related the temporal increase of the radial growth of the fungal colony. Each value is the mean of three replications (petri dishes). Means in a column followed by an asterisk are significantly different (*P*<0.05) than the mean for the corresponding wild-type isolate according to Dunnett’s test.

c Disease was assessed on a 0 to 4 severity scale based on the percentage of affected foliar tissue (0 = 0%, 1 = 1 to 33%, 2 = 24 to 66%, 3 = 67 to 100%, and 4 = dead plant) at 2–3 days intervals for 42 days growing inside a walk-in growth chamber adjusted at 25±2°C, 60–90% of relative humidity and y a 14-h photoperiod of fluorescent white light at 360 µE m^–2^ s^–1^. Incidence and severity data were used to calculate a Disease Intensity Index (DII). DII_final_: DII at the final date of disease assessment; SAUDIIPC = area under disease intensity index progress curve standardized by duration time in days of the epidemic; IP: Incubation period, estimated as the number of days to initial symptoms. Data are the means of four replicated pots, each with four plants. Means in a column followed by an asterisk are significantly different (P<0.05) than the mean for the corresponding wild-type isolate according to Dunnett’s test.

d No disease symptoms observed.

### Cytological Characterization of Compatible and Incompatible Interactions via Confocal Laser Microscopy

**Figure 1:**
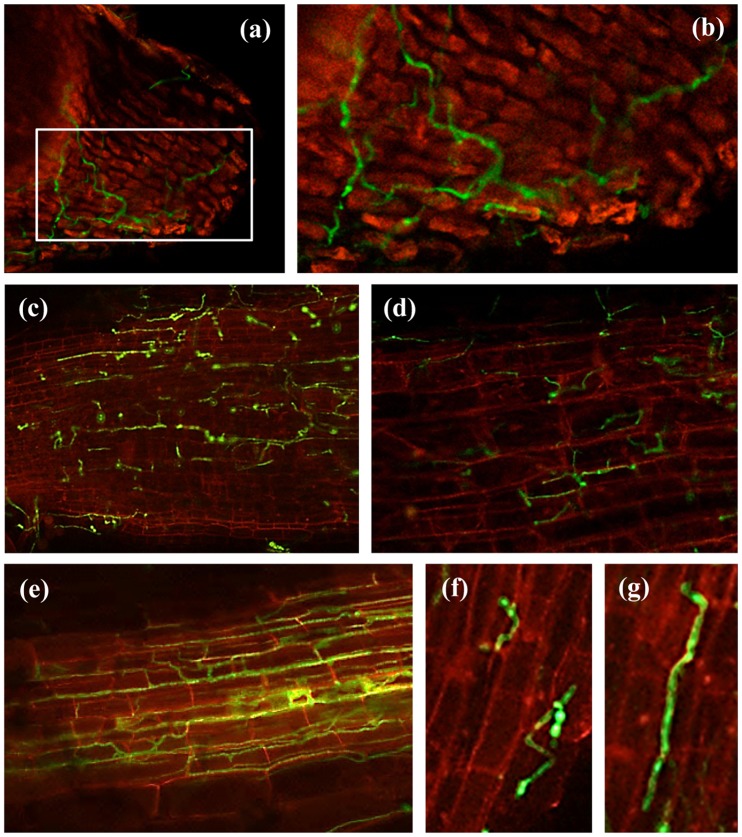
Early stages of chickpea root infection by *Fusarium oxysporum* f. sp. *ciceris* races 0 and 5 in compatible and compatible interactions. (**a, b**) germinated conidia on the root apex with primary mycelia at 1 dai; (**c–d**) primary mycelia and initial hyphal colonization on lower root zone at 2 dai; (**e**) intermediate root zone showing hyphal colonization with mycelium extending from the epidermis into cortical tissues at 6 dai; (**f**) conidia on the root surface with germ tube(s) at 1 dai; (**g**) germinated conidia on the root surface with primary mycelia at 1 dai. The *Fusarium oxysporum* f. sp. *ciceris* isolates were transformed with the ZsGreen fluorescent protein and imaged using confocal laser scanning microscopy. dai: number of days after inoculation.

**Figure 2:**
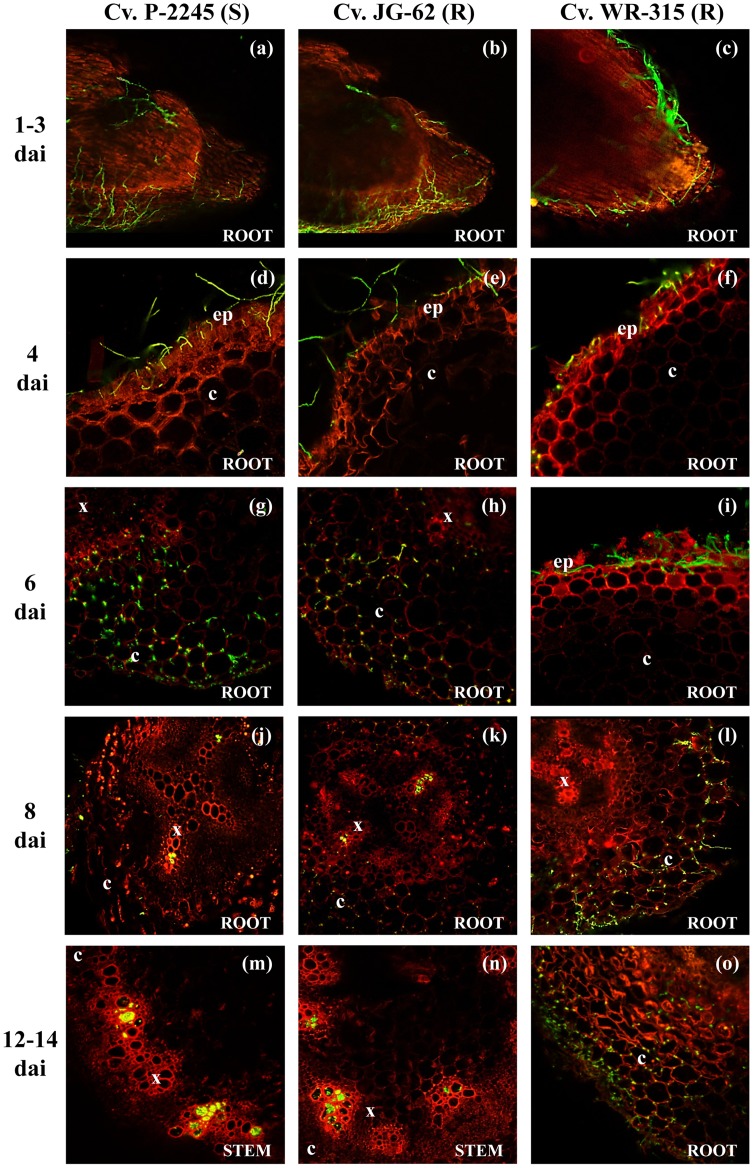
Temporal and spatial patterns of chickpea infection by *Fusarium oxysporum* f. sp. *ciceris* race 0. Colonization of three cultivars, including P-2245 (susceptible, S), JG-62 (resistant, R), and WR-315 (R) was imaged using confocal laser scanning microscopy. ep: epidermis, c: cortex, x: xylem, dai: number of days after inoculation. (**a–c**): root apex; (**d–i**): lower root zone; (**j**): upper root zone; (**k**): intermediate root zone; (**l**): lower root zone; (**m**): stem fifth internode; (**n**): stem fourth internode; (**o**): half root zone. The *Fusarium oxysporum* f. sp. *ciceris* isolate was transformed with the ZsGreen fluorescent protein.

**Figure 3:**
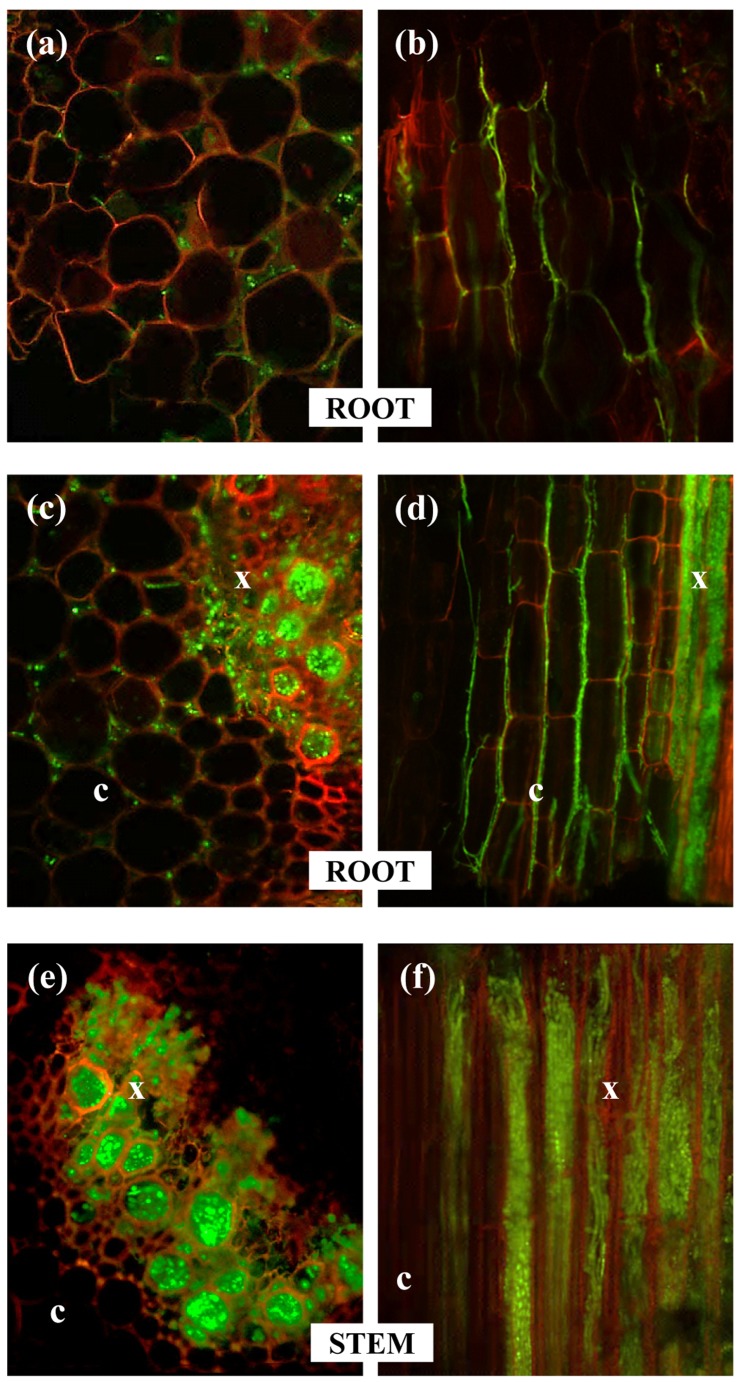
Advanced stages of chickpea infection by *Fusarium oxysporum* f. sp. *ciceris* races 0 and 5 in compatible and compatible interactions. **Left panels**: cross sections; **Right panels**: longitudinal sections. (**a,b**) intercellular colonization of cortical root tissue occurring in all compatible and incompatible interactions; (**c–d**) intercellular colonization of cortical root tissue and fungal mycelia through root xylem vessels occurring in all compatible interactions and in the incompatible interactions of cv. JG-62/race 0 and cv. WR-315/race 5; (**e–f**) heavy colonization of xylem stem vessels occurring in all compatible interactions and in the incompatible interaction of cv. JG-62/race 0. The *Fusarium oxysporum* f. sp. *ciceris* isolates were transformed with the ZsGreen fluorescent protein and imaged using confocal laser scanning microscopy. c: cortex, x: xylem.

**Figure 4:**
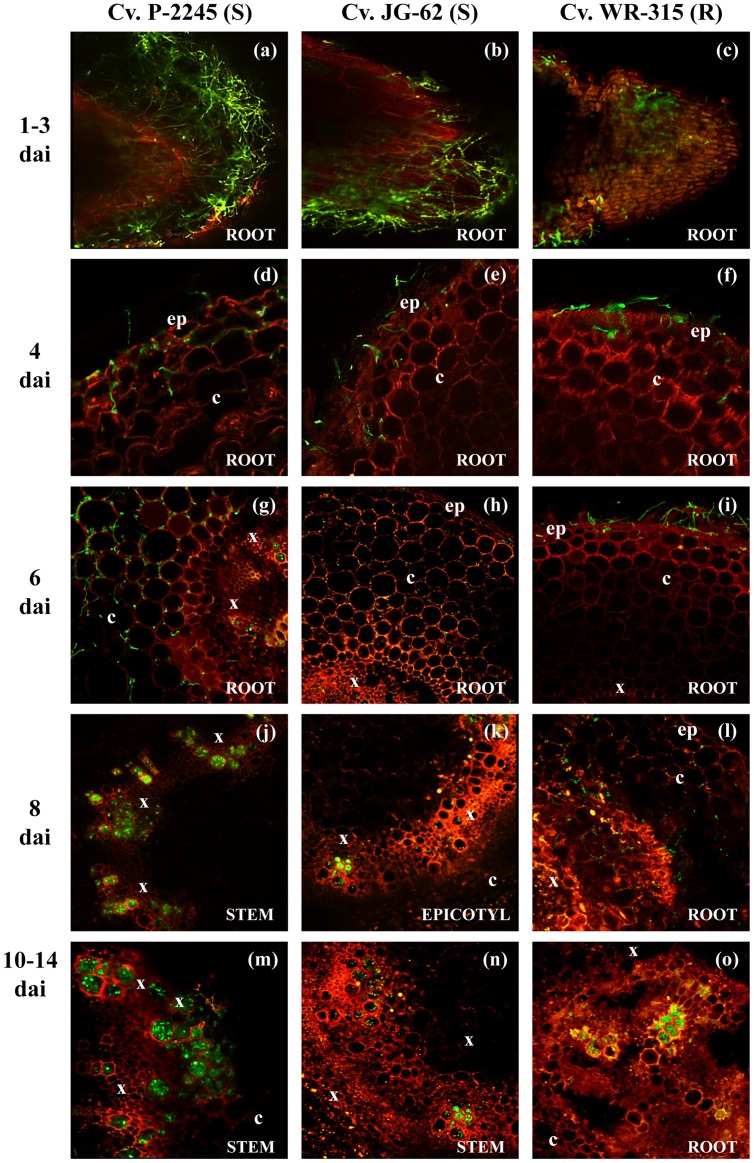
Temporal and spatial patterns of chickpea infection by *Fusarium oxysporum* f. sp. *ciceris* race 5. Colonization of three cultivars, including P-2245 (susceptible, S), JG-62 (S), and WR-315 (resistant, R) was imaged using confocal laser scanning microscopy. ep: epidermis, c: cortex, x: xylem, dai: number of days after inoculation. (**a–c**): root apex; (**d–i**): lower root zone; (**j**): stem second internode; (**k**): epicotyl; (**l**): lower root zone; (**m**): stem fourth internode; (**n**): stem third internode; (**o**): lower root zone. The *Fusarium oxysporum* f. sp. *ciceris* isolate was transformed with the ZsGreen fluorescent protein.

No substantial differences were observed during the initial stages of infection of the tap root and root hairs by races 0 and 5 (Fig. 1). However, the spatial-temporal dynamics of subsequent infection processes of chickpea plants varied considerably among the *F. oxysporum* f. sp. *ciceris* race/chickpea cultivar combinations studied here (Figs. 1, 2, 3, 4, Table 2).

**Table 2:** Colonization process of different parts of three chickpea cultivars showing differential resistance by *Fusarium oxysporum* f. sp. *ciceris* races 0 and 5^a^.

Time (days after inoculation)	Cultivar		
	P-2245	JG-62	WR-315
***F. oxysporum*** ** f. sp. ** ***ciceris*** ** race 0**			
1 to 3	Root (L): epidermis	Root (L): epidermis	Root (L): epidermis
4	Root (L): epidermis	Root (L): epidermis	Root (L): epidermis
6	Root (L): cortex	Root (L): cortex	Root (L): epidermis
8	Root (U): xylem vessels	Root (L): xylem vessels	Root (L): cortex
10	Stem (L): xylem vessels	Root (U): xylem vessels	Root (M): cortex
12	Stem (M): xylem vessels	Stem (M): xylem vessels	Root (M): cortex
14	Stem (U): xylem vessels	Stem (M): xylem vessels	Root (M): cortex
***F. oxysporum*** ** f. sp. ** ***ciceris*** ** race 5**			
1 to 3	Root (L): epidermis	Root (L): epidermis	Root (L): epidermis
4	Root (L): cortex	Root (L): cortex	Root (L): epidermis
6	Root (L): xylem vessels	Root (M): cortex	Root (L): epidermis
8	Stem (L): xylem vessels	Epicotyl: xylem vessels	Root (L): cortex
10	Stem (M): xylem vessels	Stem (L): xylem vessels	Root (L): cortex
12	Stem (U): xylem vessels	Stem (M): xylem vessels	Root (L): xylem vessels
14	Stem (U): xylem vessels	Stem (U): xylem vessels	Root (L): xylem vessels

a Progress of pathogen proliferation along the vertical axis of the plant was analyzed in the following parts: Root: (L) lower: meristem, elongation and differentiation zones; (M) medium, (U) upper. Stem: (L) lower, internode 1 to 2^nd^; (M) medium, internode 3 to 4^th^; (U) upper, 5^th^ internode or above.

For race 0, the earliest infection event consisted of the development of a dense hyphal network without pattern on the surface of the tap root, including the root cap, apical meristem and subapical zones, which took place during the first 4 days after inoculation (dai) (Figs. 1a,b, 2a–c, Table 2). The fungus also grew on the surface of lateral roots in a manner similar to that observed on the taproot with the apical zone being heavily colonized by hyphae that grew along anticlinal wall junctures of epidermal cells (Fig. 1c–e). Following the surface growth, the hyphae directly penetrated epidermal cells without forming specialized penetration structures (Figs. 1f,g, 2d–f). By 6 dai, the race 0 transformant was found in the intercellular spaces of the outermost cell layers of the root cortex of cvs. P-2245 (compatible interaction) and JG-62 (incompatible interaction) (Figs. 2g–h, 3a,b), but it remained restricted within the root epidermis of cv. WR-315 (incompatible interaction) (Fig. 3i, Table 2). Two days later, the intercellular colonization of the root cortex was extensive in the susceptible cv. P-2245, and the pathogen reached the central root cylinder and entered into the xylem vessels of the upper root zone (Figs. 2j, 3c,d). Comparatively, the resistant reaction was characterized by restricted pathogen growth within the root tissues, but the degree of restriction varied between the two cultivars. By 8 dai the race 0 transformant remained restricted to intercellular spaces of the root cortex of ‘WR315’ (Fig. 2l) or could reach a few individual vessels in the lowermost root zone of ‘JG-62’ (Fig. 1k). Based on those observations, the root apex was identified as the preferential court of infection for the three chickpea cultivars by race 0, regardless their resistance (Fig. 2a–c).

By 10 dai, the race 0 transformant progressed upward to reach the hypocotyl, epicotyl, and lower stem zone of susceptible ‘P-2245’ seedlings. The first disease symptoms (i.e. yellowing of foliage at the stem base characteristic of the yellowing syndrome caused by race 0) was observed at 10 dai. Thereafter, the pathogen colonized the upper stem vascular tissue, reaching the fifth internode by 12 dai (Figs. 2m, 3e,f). By 16 dai, all ‘P-2245’ plants were severely affected, eventually resulting in plant senescence and decay. At this stage, fungal hyphae erupted from the stem vascular tissue into the surrounding cortical parenchyma.

In race 0-resistant ‘JG-62’ chickpea plants, the pathogen progressed upwards along the stem axis at a rate slower than that observed in the susceptible cv. P-2245. It took 12 dai for the pathogen to reach the lower stem xylem vessels (Fig. 2n). The pathogen grew up to reach the fourth stem internode, although no symptoms were observed during the entire duration of the experiment. Comparatively, in the race 0-resistant ‘WR-315’ plants, the pathogen remained restricted within the intercellular spaces of the cortex in the intermediate root zone, but it was never found in the vascular system (Fig. 2o). Infected plants remained free of symptoms throughout the duration of the experiment.

Early infection events of susceptible cvs. P-2245 and JG-62 by a transformant of the highly virulent *F. oxysporum* f. sp. *ciceris* race 5 were similar to those described for *F. oxysporum* f. sp. *ciceris* race 0 (Fig. 1, Table 2). From 1 to 4 dai, extensive growth of the fungus on the root surface and the root meristem was observed (Fig. 4a–c). Thereafter, growth of the race 5 transformant within plants of these two cultivars progressed faster compared with that of race 0 in cv. P-2245 (Fig. 4d–f). Colonization of xylem vessels in the apical root zone of cv. P-2245 by the race 5 transformant started as early as 6 dai and was preceded by extensive intercellular growth within the root cortex (Fig. 4g). Comparatively, no vascular infection was noticed in roots of cvs. WR-315 (incompatible interaction) and JG-62 (compatible interaction) at that same time, and the pathogen appeared restricted within the epidermis and root cortex of the apical root zone, respectively (Fig. 4h,i). However, by 8 dai hyphae of the race 5 transformant reached the stem xylem vessels and were located in the epicotyl and second stem internode of susceptible cvs. JG-62 (Fig. 4k) and P-2245 (Fig. 4j), respectively, but no symptoms were observed. Symptoms started to develop on day 9 dai, at which time the pathogen hyphae within the xylem vessels had reached up to the fourth and third stem internodes of ‘P-2245’ and ‘JG-62’ plants, respectively (Fig. 4m,n). Symptoms on these two cultivars consisted of severe leaf chlorosis and flaccidity characteristic of the wilting syndrome. Growth of the pathogen within the stem xylem continued, subsequently reaching the uppermost stem tissues in ‘P-2245’, and the 3 to 4^th^ internodes of ‘JG-62’ by 12 dai. Two days later, the entire vascular system was colonized by the pathogen, and the plants died. Conversely, plants of the resistant cv. WR-315 remained symptomless throughout the duration of the experiment, though race 5 was able to invade the xylem vessels at the apical root zone by 12 dai, and occasionally it was found in the hypocotyl xylem vessels by 18 dai. *F. oxysporum* f. sp. *ciceris* race 5 was found neither in epicotyl nor in stem tissues (Fig. 4o).

Colonization by the pathogen was quantitatively assessed using the incidence (proportion of colonized vessels, IcVC) and intensity (lumen area of the transverse section of an infected vessel occupied by fungal hyphae, ItVC) parameters. These two variables were highly and positively correlated (*r = *0.7936, *P*<0.0001); therefore, only results concerning data analysis of ItVC are shown. Overall, the ItVC was significantly influenced (*P*<0.05) by the experimental treatment combinations and their interactions. However, most ItVC variation was accounted by the main factors in the study, i.e., pathogenic race [*F = *500.81, *P*<0.0001, 46.16% Mean Square Error (MSE)] and chickpea cultivar (*F = *279.16, *P*<0.0001, 25.73% MSE). Variation in ItVC was accounted to a much lesser extent by sampling time (*F = *75.13, *P*<0.0001, 6.93% MSE) and sampled plant zone (*F = *16.88, *P*<0.0001, 1.56% MSE). Interactions among main factors were statistically significant (*P*<0.05) in some cases, but they explained a marginal proportion of the variation in ItVC, which ranged from 5.59 to 0.67% of the MSE (data not shown).

**Figure 5:**
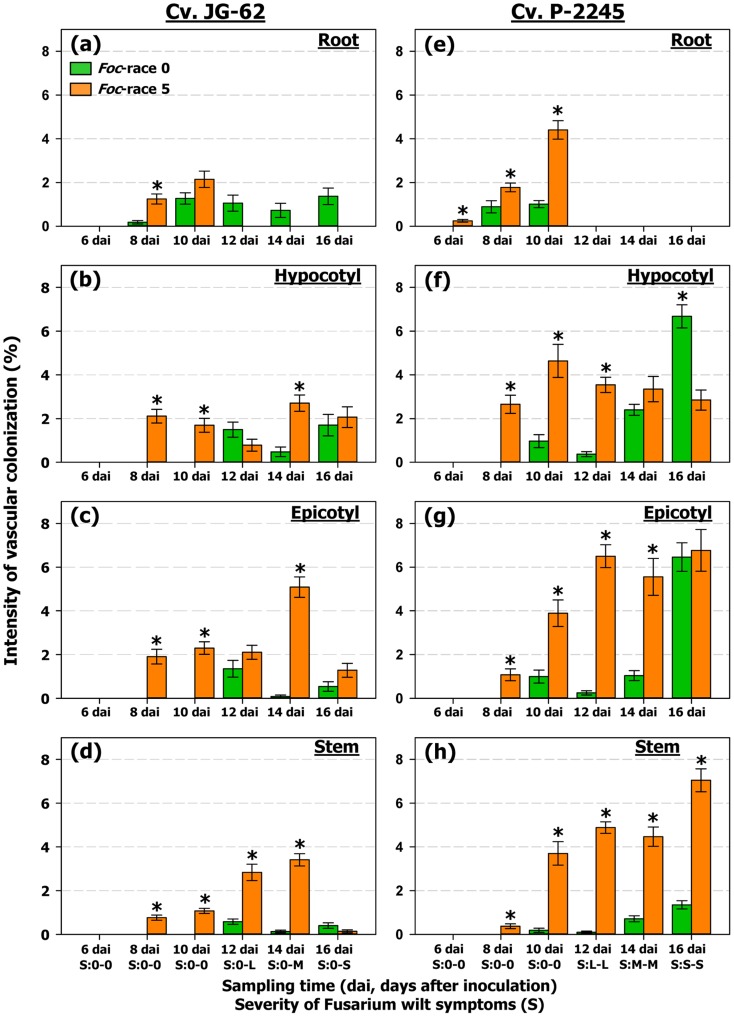
Intensity of vascular colonization (ItCV) by races 0 and 5 of *Fusarium oxysporum* f. sp. *ciceris*. The *Fusarium oxysporum* f. sp. *ciceris* (*Foc*) isolates were transformed with the ZsGreen fluorescent protein. Plants were sampled daily from 1 to 4 days after inoculation (dai), and at 2 days intervals from 6 to 18 (dai). Tissues sampled included: Root: lower and intermediate zone of the tap root (**a, e**); Hypocotyl (**b, f**) and epicotyl (**c, g**): zones immediately before and after the insertion of cotyledons, respectively; Stem (**d, h**): internodes one to fifth of the main stem. Severity (S) indicate the disease severity (DS) level of foliar symptoms assessed at each sampling time using a 0 to 4 scale based on the percentage of affected foliar tissue (0 = 0%, 1 = 1 to 33%, 2 = 24 to 66%, 3 = 67 to 100%, and 4 = dead plant). S = 0: no symptoms; S = L: initial symptoms (0<DS<2); S = M: moderate symptoms (2<DS≥3); and S = S: severe symptoms (DS≥3), for *Foc*-0 (first character) and *Foc*-5 (second character). There were four plants (two plastic pots) per *F. oxysporum* f. sp. *ciceris* race/chickpea cultivar combination analysed for each sampling time. The experiments were arranged in a randomized complete blocks design, and were repeated twice. For each sampled tissue, vascular colonization was assessed on four blocks with 20 cells (xylem vessels) per block, covering the entire vascular cylinder. Error bars indicate the standard error of the mean. Significant differences (*P*<0.05) between ItCV level reached on races 0 and 5 for each chickpea cultivar/tissue combination at each sampling date are indicated by an asterisk.

The ItVC level tended to increase over time, reaching the highest value at 16 dai, except for the cv. JG-62/race 5 combination (Fig. 5). In this combination, ItVC decreased in plants sampled at 16 dai probably due to tissue disintegration and death of plant (i.e., disease severity rating 3 to 4 in the 0–4 scale) (Fig. 5a–d). The mean level of ItVC was significantly higher (*F = *170.94, *P*<0.0001) at sampling times in which plants showed disease symptoms. Moreover, ItVC values were positively and significantly correlated with the disease severity ratings (*r = *0.5777, *P = *0.0120) (data not shown).

**Figure 6:**
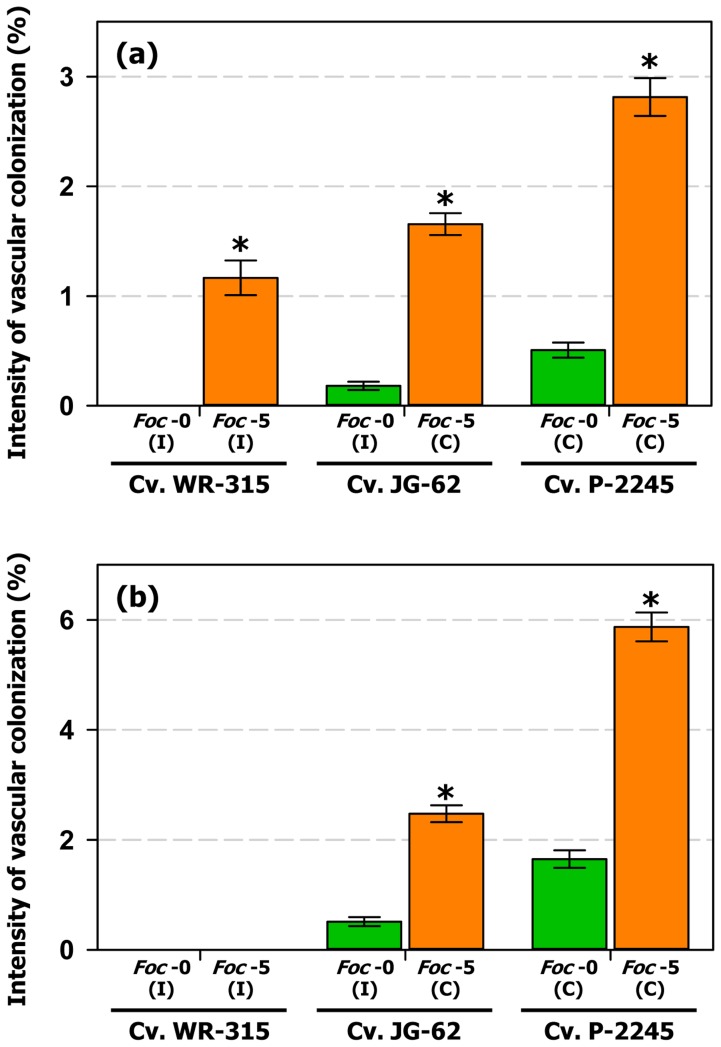
Intensity of vascular colonization (ItCV) by races 0 and 5 of *Fusarium oxysporum* f. sp. *ciceris* at early and advanced stages of plant colonization. (**a**) ItCV mean values at early stages of plant colonization, i.e., sampling times in which no disease symptoms develop in the compatible interactions. (**b**) ItCV mean values at advanced stages of plant colonization, i.e., at sampling times in which symptoms develop in compatible interactions. The *Fusarium oxysporum* f. sp. *ciceris* (*Foc*) isolates were transformed with the ZsGreen fluorescent protein. I: incompatible interaction; C: compatible interaction. Plants were sampled daily from 1 to 4 days after inoculation (dai), and at 2 days intervals from 6 to 18 (dai). Tissues sampled included: Root: lower and intermediate zone of the tap root; Hypocotyl and epicotyl: zones immediately before and after the insertion of cotyledons, respectively; Stem: internodes one to fifth of the main stem. There were four plants (two plants per pot) per *F. oxysporum* f. sp. *ciceris* race/chickpea cultivar combination analysed for each sampling time. The experiments were arranged in a randomized complete blocks design, and were repeated twice. For each sampled tissue, vascular colonization was assessed on four blocks with 20 cells (xylem vessels) per block, covering the entire vascular cylinder. Error bars indicate the standard error of the mean. Significant differences (*P*<0.05) between ItCV level reached at races 0 and 5 for each chickpea cultivar are indicated by an asterisk.

At early stages of plant colonization, i.e., at sampling times in which no disease symptoms develop in the compatible interactions, ItCV was significantly higher (*F = *185.21, *P*<0.0001) on compatible interactions (Fig. 6a). Among them, ItVC was highest (2.81±0.17%; *F = *169.04, *P*<0.0001) in the most conducive P-2245/race 5 interaction, decreased significantly (1.66±0.10%; *F = *56.69, *P*<0.0001) in cv. JG-62 infected by this same race 5, and was lowest (0.51±0.07%; *F = *168.03, *P*<0.0001) in plants of cv. P-2245 infected by race 0 (Fig. 6a). Among incompatible interactions, ItVC was highest on the cv. WR-315/race 5 interaction (1.17±0.16%; *F = *65.18, *P*<0.0001) and decreased to 0.18±0.04% (*F = *40.96, *P*<0.0001) in ‘JG-62’ plants infected by race 0. There was no invasion of xylem vessels in the cv. WR-315/race 0 interaction (Fig. 6a).

At advanced stages of plant colonization, i.e., in sampling times in which disease symptoms develop in compatible interactions, infection of xylem vessels only occurred in ‘JG-62’ plants infected by race 0 that showed a mean ItCV of 0.51±0.08% (Fig. 6b). In compatible interactions, the mean ItVC was highest (*F = *310.85, *P*<0.0001) for the cv. P-2245/race 5 interaction with a mean ItVC of 5.87±0.26%, followed by that in cv. JG-62 infected by the same race 5 (2.48±0.15%; *F = *286.34, *P*<0.0001) (Fig. 6b). Comparatively, infection of those two cultivars by the less virulent race 0 was associated with significantly lower ItVC values, i.e., 1.65±0.16% in cv. P-2245 (*F = *185.40, *P*<0.0001) and 0.51±0.08% in the incompatible cv. JG-62/race 0 interaction (*F = *20.82, *P*<0.0001) (Fig. 6b). It should be emphasized that, as it was pointed out before, no symptoms developed in ‘JG-62’ infected by race 0.

Variation of mean ItVC along the main axis of a plant differed considerably among chickpea cultivar-pathogenic race experimental combinations, sampling times and the severity of symptoms (Fig. 5; Table 3). Thus, for asymptomatic plants in the incompatible cv. JG-62/race 0 interaction, the mean ItVC in root and hypocotyl was significantly higher (*F = *14.15, *P = *0.0002) than in epicotyl and stem (Fig. 5a–d), whereas the mean ItVC values in the epicotyl were similar to those in the stem (*F = *1.72, *P = *0.1900) (Fig. 5c,d; Table 3). Overall, the mean ItVC was similar (*F*<2.21, *P*>0.1385) in subterranean and aerial plant parts of the compatible cv. JG-62/race 5 interaction that were sampled at the same time that those in the incompatible cv. JG-62/race 0 interaction that remained asymptomatic (Fig. 5a–d). However, ItVC in the epicotyl was significantly higher (*F = *18.05, *P*<0.0001) when compared with that in the stem (Fig. 5c,d; Table 3). Nevertheless, at later sampling times, with plants showing Fusarium wilt symptoms, the mean ItVC increased from bottom to top in a plant. Thus, the aerial plant tissues showed a higher colonization intensity (*F = *6.43, *P = *0.0144) compared with that in subterranean tissues, with ItVC being highest (*F*<11.70, *P*<0.0007) in the epicotyl (Fig. 5a–d; Table 3). However, this colonization pattern did not hold for plants sampled at 16 dai. At this time, severe symptoms in a plant were associated with extensive collapse and xylem degeneration, particularly in the aerial tissues, which determined the occurrence of ItVC means lower than those in previous sampling times (Fig. 5c,d).

**Table 3:** Effects of sampling time and plant tissue on vascular colonization intensity by *Fusarium oxysporum* f. sp. *ciceris* (*Foc*) races 0 and 5 in chickpea cultivars showing differential resistance.

	Cultivar JG-62				Cv. P-2245			
	*Foc*-0		*Foc*-5		*Foc*-0		*Foc*-5	
Source of variation^a^	*F*	*P*>*F*	*F*	*P*>*F*	*F*	*P*>*F*	*F*	*P*>*F*
Sampling time	15.96	<0.0001	30.20	<0.0001	165.68	<0.0001	30.72	<0.0001
Plant zone	9.22	<0.0001	7.10	<0.0001	37.00	<0.0001	5.60	0.0008
Sampling time × Plant zone	2.57	0.0022	7.69	<0.0001	20.77	<0.0001	6.47	<0.0001
Contrasts for asymptomatic plants (sampling times 8 and 10 dai) b								
Subterranean vs. aerial tissues	–c	–	2.20	0.1386	10.07	0.0016	11.56	0.0007
Root vs. Hypocotyl	–	–	0.55 d	0.4578	6.09	0.0139	1.45	0.2283
Hypocotyl vs. Epicotyl	–	–	0.50	0.4816	<0.01	0.9525	6.37	0.0118
Epicotyl vs. Stem	–	–	18.05	<0.0001	4.58	0.0328	0.93	0.3347
Contrasts for symptomatic plants (sampling times 12 to 16 dai) b								
Subterranean vs. aerial tissues	14.15	0.0002	6.43	0.0114	44.53	<0.0001	39.71	<0.0001
Hypocotyl vs. Epicotyl	6.77	0.0095	11.69	0.0007	4.78	0.0291	39.67	<0.0001
Epicotyl vs. Stem	1.72	0.1900	5.99	0.0147	51.61	<0.0001	2.83	0.0928

a Plants were grown hydroponically in a growth chamber at 25±2°C and a 14-h photoperiod of fluorescent white light of 360 µE m^−2^ s^−1^ in an orbital shaker at 120 rpm. Plants were sampled at 2 days intervals from 6 to 18 days after inoculation (dai). Tissues sampled included: Root: lower and intermediate zone of the tap root; Hypocotyl and epicotyl: zones immediately before and after the insertion of cotyledons, respectively; Stem: internodes one to third of the main stem. Four plants (two plastic vessels) per *F. oxysporum* f. sp. *ciceris* race/chickpea cultivar combination were analysed at each sampling time. The experiments were arranged in a randomized complete blocks design, and were repeated twice. For each sampled tissue, vascular colonization was assessed on four blocks with 20 cells (xylem vessels) per block, covering the entire vascular cylinder.

b Linear single-degree of freedom contrasts were computed to test the effect of selected experimental treatment combinations at *P*<0.05.

c Experimental combinations in which no disease symptoms developed.

d Underlined values indicate that estimated value for the first term of the contrast was lower than that of the second term.

In compatible interactions of the highly susceptible cv. P-2245 with the two races, the pattern of quantitative colonization during the time period that plants were asymptomatic was similar for races 0 and 5, with the mean ItVC being higher (*F*>10.06, *P*<0.0017) in the subterranean tissues compared to that in aerial tissues (Table 3), though mean values for race 5 were significantly higher than those for race 0 (*F = *171.31, *P*<0.0001) (Fig. 5e–h). Conversely, that pattern of colonization differed much between races when infection gave rise to the symptomatic stage (Fig. 5). Thus, for *F. oxysporum* f. sp. *ciceris* race 0 the ItVC was significantly higher (*F = *44.53, *P*<0.0001) in subterranean than in aerial plant parts (Fig. 5e–h; Table 3), and ItVC decreased in the upper stem tissues as the severity of symptoms increased (*F = *51.61, *P*<0.0001) (Fig. 5h; Table 3). On the contrary, for race 5 ItVC was higher (*F = *39.71, *P*<0.0001) in the aerial than in subterranean plant parts, reaching similar values (*F = *2.83, *P = *0.0928) in the epicotyl and upper stem tissues (Fig. 5e–h; Table 3).

## Discussion

This study demonstrates for the first time the utility of CLSM for better understanding the spatial-temporal dynamics of the infection and colonization processes of *F. oxysporum* f. sp. *ciceris* races 0 and 5 in three chickpea cultivars including compatible and incompatible interactions.

The use of genetically transformed organisms requires prior demonstration that transformation did not modify any biological feature of the wild strains. Phenotypic characterization of the transformants developed in our study, including growth rate and morphology, pathogenicity and virulence on a susceptible chickpea cultivar, and pathogenic race, showed that most of them retained the biological features of the wild-type strain. Based on these evaluations, two transformants were selected for the study: F11 and F93 derived, respectively from wild strains Foc-7802 (race 0) and Foc-W6-1 (race 5).

We qualitatively and quantitatively assessed the interactions of *F. oxysporum* f. sp. *ciceris* races 0 and 5 with chickpea cultivars in both compatible and incompatible interactions from the early stages of root colonization to infection and extensive stem vascular colonization. In contrast to our work, most studies on formae speciales of *F. oxysporum*/host plant interactions involving the use of CLMS were restricted to subterranean host tissues [e.g., 18,19,33,34,35,36,37], with only a few of them trying to observe colonization in the stem tissues [20], [21], [38].

Ingress of formae speciales of *F. oxysporum* into host plants was assumed to take place either by direct penetration or through wounds [39]. In this present study, penetration of chickpea roots by *F. oxysporum* f. sp. *ciceris* races 0 and 5 occurred through intact tissues without need of differentiating specialized penetration structures. This confirms previous observations in this same pathosystem by Jiménez-Díaz *et al.* [23], as well as most works performed in other Fusarium wilts [e.g., 17,18,21,39,40,41,42,43]. In our study, the meristematic cells of chickpea root apex were identified as the preferential infection site for *F. oxysporum* f. sp. *ciceris*. This does not agree with previous studies on this pathosystem, which concluded that the cotyledons and zones of the epicotyl and hypocotyl at the junction of or close to cotyledons are preferential invasion sites of the pathogen [23], [24]. Differences in the inoculation methods used may account for differences between these studies. Indeed, Olivain & Alabouvette [43] observed that colonization of the root elongation zone and root apex were the preferential infection sites of tomato roots inoculated with *F. oxysporum* f. sp. *lycopersici* by the hydroponic culture method, but that did not hold when plants were inoculated by the soil-infestation inoculation method [22], [35].

In the absence of wounds, most formae speciales of *F. oxysporum* can get ingress into the host through a variety of root tissues, including the root meristem, root hairs, and different zones of the root and seed [39]; however, most reports support the root tip as a preferential site for host invasion by these pathogens. This was reported for *F. oxysporum* f. sp. *asparagi* in asparagus [44], *F. oxysporum* f. sp. *dianthi* in carnation [45], *F. oxysporum* f. sp. *lini* in flax [46], *F. oxysporum* f. sp. *pini* in pine seedlings [47], *F. oxysporum* f. sp. *vanillae* in vanilla [48], and *F. oxysporum* f. sp. *vasinfectum* in cotton [42]. The lack of penetration through the host root apex was described only for *F. oxysporum* f. sp. *cucumerinum* in cucumber [49]. Others reported that host penetration sites for formae speciales of *F. oxysporum* include the zone of root elongation by *F. oxysporum* f. sp. *lentis* in lentils [50], and the cotyledonary node and undifferentiated region of the root tip for *F. oxysporum* f. sp. *pisi* in peas [51]. In our study, the rate and intensity at which the pathogen colonized the epicotyl and stem xylem was directly related to the degree of compatibility of the *F. oxysporum* f. sp. *ciceris* race/chickpea cultivar combination. Thus, both in the root and stem the fastest and higher intensity of xylem colonization (ItVC) occurred in the compatible interactions. Thus, ItCV was highest in cv. P-2245 infected by the highly virulent race 5, followed by that in cv. JG-62 infected by the same race and in cv. P-2245 infected by the less virulent race 0. This quantitative pattern of xylem colonization agrees with observations by García-Sánchez *et al.* [20] in bean infected with *F. oxysporum* f. sp. *phaseoli*, in which the rate and intensity of colonization of xylem vessels were higher for the most virulent isolate of the pathogen. The higher biomass of race 5 compared with that of race 0 observed and quantified with CLSM confirms results from our previous studies using real-time quantitative PCR, in which the quantity of race 5 DNA both in subterranean and aerial plant tissues was significantly higher than that of race 0 [52].

Results of particular interest from this present study are those concerning the incompatible interactions. In general, asymptomatic reactions in fungal wilt pathosystems are associated with the absence of vascular invasion by wilting fungi. In our study, that occurred only for the cv. WR-315/race 0 combination. However, when this same cultivar was infected by the more virulent race 5, and particularly when cv. JG-62 was infected by the less virulent race 0, the pathogen either invaded the root xylem vessels to a limited degree or extensively colonized the root and stem xylem vessels, respectively, but without any symptom’s development. This observed lack of vascular colonization in highly incompatible chickpea/*F. oxysporum* f. sp. *ciceris* interactions agrees with reports from other authors that used light microscopy for observations of paraffin-embedded tissues [23], [24]. Stevenson *et al.* [24] failed to find the fungus in the xylem vessels of any of the resistant cvs. WR-315 and CPS-1 inoculated with each of races 1A or 2, and Jiménez-Díaz *et al.* [23] reported the absence of fungal structures in root and stem tissues of resistant ‘WR-315’ inoculated with either race 0 or 5, and resistant ‘JG-62’ inoculated with race 0. However, these later authors also reported positive isolation of the pathogen in artificial growth media from subterranean tissues located at or near sites of cotyledon attachment. From this, Jiménez-Díaz *et al.* [23] concluded that *F. oxysporum* f. sp. *ciceris* can remain associated with those tissues from which it could be isolated, though to such a minor extent that no fungal structures could be observed microscopically. Our results for the incompatible cv. WR-315/race 5 interaction are consistent with the observations by those authors and demonstrate the high efficiency of the CLSM technology to visualize and characterize in vivo host-pathogen interactions.

The difference in the degree of colonization of resistant cultivars by *F. oxysporum* f. sp. *ciceris* races found in this present study was also reported for other formae speciales of *F. oxysporum* in resistant cultivars of different hosts. Fang *et al.* [38] indicated that *F. oxysporum* f. sp. *fragariae* remained confined in the epidermal layer of the root in a resistant strawberry cultivar, and Tessier *et al.* [53] described that *F. oxysporum* f. sp. *pisi* was restricted to the initially infected root vessels in asymptomatic reactions of a resistant pea cultivar. Conversely, Zvirin *et al.* [21] reported vascular colonization of a resistant melon cultivar by *F. oxysporum* f. sp. *melonis* race 1.2, although that occurred at a lower rate and incidence than that in the susceptible cultivar. Similarly, Ramírez-Suero *et al.* [19] indicated that there were no significant differences between the degree of colonization of susceptible and tolerant *M. truncatula* genotypes by *F. oxysporum* f. sp. *medicaginis*.

Less common among Fusarium wilt diseases is the occurrence of extensive vascular colonization of the plant axis in the absence of visible symptoms, as observed in this study for cv. JG-62 infected with race 0. On the contrary, the degree of symptom expression is usually reported as being closely related to the extent of vascular colonization by the pathogen [54], as it was found in the compatible chickpea/*F. oxysporum* f. sp. *ciceris* interactions in this present study. Interestingly, cv. JG-62 is susceptible to all known races of the pathogen causing the wilting syndrome but shows a moderate reaction to race 1B/C, which together with race 0, belongs to the yellowing pathotype [55]. The asymptomatic response of ‘JG-62’ plants to extensively xylem colonization by race 0 corresponds to a tolerant reaction rather than true resistance. This could indicate that either the fungal biomass has not yet reached the threshold for inducing symptoms or, more likely, that other mechanisms together with the presence of pathogen in the vascular tissues are required for the disease to develop.

The differential patterns of infection and colonization of chickpea cultivars by different races could be related to differences in the defence mechanisms between resistant and susceptible cultivars. In fact, García-Limones *et al.* [56] indicated that infection by *F. oxysporum* f. sp. *ciceris* race 5 caused significant changes in activity levels of several oxidative stress-related enzymes in the apoplast of chickpea roots, although there were differences between susceptible (‘JG-62’) and resistant (‘WR-315’) cultivars. Thus, while infection by the pathogen increased apoplastic glutathione reductase (GR) activity and decreased superoxide dismutase (SOD) and diamine oxidase (DAO) activities in the susceptible and resistant cultivars, that occurred earlier (i.e., GR and SOD) or in higher quantities (i.e., DAO) in the resistant (‘WR-315’) than in the susceptible (‘JG-62’) cultivar. Conversely, the main specific responses to infection in each interaction were an increase in apoplastic ascorbate peroxidase activity in cv. WR-315 and a decrease of this activity in cv. JG-62.

Finally, the results in our study are relevant for disease control. It is well accepted that the use of resistant cultivars is one of the few and most effective and environmentally friendly control measures of Fusarium wilt of chickpeas [4], [57], [58]. However, populations of *F. oxysporum* f. sp. *ciceris* display high pathogenic variability, which limits the effectiveness and extensive use of available resistance and makes necessary the adequate characterization of resistance of chickpea lines and cultivars to specific races of *F. oxysporum* f. sp. *ciceris* [52], [57], [59], [60]. Our work demonstrates the utility of CLSM as a rapid and efficient method to qualify and quantify infection by *F. oxysporum* f. sp. *ciceris* races in plant roots and stem, which allows for the accurate assessment of disease reactions of chickpea germplasm particularly in incompatible interactions. This technique allows selecting breeding lines with complete resistance or those that being partially resistant may carry traits of agronomic and/or commercial interest. Thus, alone or in combination with the q-PCR protocol developed in a previous study [52], use of CLSM is a powerful tool for studies aimed at gaining better understanding of chickpea - *F. oxysporum* f. sp. *ciceris* interactions and the efficient management through disease resistance breeding in Fusarium wilt of chickpeas.
